# Reframing Psychiatry for Precision Medicine

**DOI:** 10.3390/jpm10040144

**Published:** 2020-09-25

**Authors:** Elizabeth B. Torres

**Affiliations:** 1 Department of Psychology, Rutgers University, New Brunswick, NJ 08854, USA; ebtorres@psychology.rutgers.edu; Tel.: +1-(011)-858-445-8909; Fax: +1-(011)-732-445-2987; 2 Center for Cognitive Science (RUCCS), Rutgers University, New Brunswick, NJ 08854, USA; 3 Computer Science, Center for Biomedicine Imaging and Modelling (CBIM), Rutgers University, New Brunswick, NJ 08854, USA

**Keywords:** autism, schizophrenia, mental depression, ataxia, fragile X, Parkinson’s disease, mitochondria, gene expression, tissues, neurological disorders, nervous systems disorders

## Abstract

The art of observing and describing behaviors has driven diagnosis and informed basic science in psychiatry. In recent times, studies of mental illness are focused on understanding the brain’s neurobiology but there is a paucity of information on the potential contributions from peripheral activity to mental health. In precision medicine, this common practice leaves a gap between bodily behaviors and genomics that we here propose to address with a new layer of inquiry that includes gene expression on tissues inclusive of brain, heart, muscle-skeletal and organs for vital bodily functions. We interrogate gene expression on human tissue as a function of disease-associated genes. By removing genes linked to disease from the typical human set, and recomputing gene expression on the tissues, we can compare the outcomes across mental illnesses, well-known neurological conditions, and non-neurological conditions. We find that major neuropsychiatric conditions that are behaviorally defined today (e.g., autism, schizophrenia, and depression) through DSM-observation criteria have strong convergence with well-known neurological conditions (e.g., ataxias and Parkinson’s disease), but less overlap with non-neurological conditions. Surprisingly, tissues majorly involved in the central control, coordination, adaptation and learning of movements, emotion and memory are maximally affected in psychiatric diagnoses along with peripheral heart and muscle-skeletal tissues. Our results underscore the importance of considering both the brain–body connection and the contributions of the peripheral nervous systems to mental health.

## 1. Introduction

**Figure 1:**
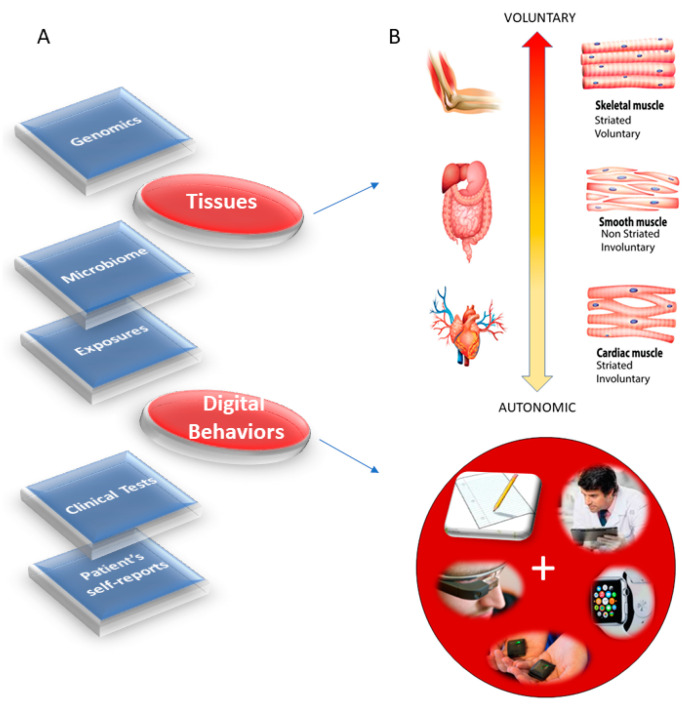
Roadmap to implement the precision medicine model for diagnoses and treatments of mental illnesses. (**A**) PM’s interconnected knowledge network can contribute information about the individual’s medical history, behaviors, environment, microbiome, and genetic makeup. Importantly, the new proposed layer of *digitized behaviors* leveraging the wearable biosensors revolution can transform medicine by creating truly personalized assessments. Additionally, the layer of behaviors can be connected to nervous system functioning via fundamental levels of neuromotor control that span along a phylogenetically orderly taxonomy. (**B**) This proposed taxonomy is based on levels of maturation in autonomous neuromotor control, linked to three fundamental muscle types: autonomic (by cardiac muscles), involuntary (by smooth muscles) and voluntary (by skeletal muscles). By linking the fundamental muscle types to the levels of control in the nervous systems, digital behaviors can then be mapped to bodily autonomy, bodily autonomy mapped to muscle types and muscle types mapped to genes/proteins. Any measure of treatment effectiveness for mental illnesses can then map back to improvements in observable behaviors embedded in activities of daily social life.

Modern medicine is at an inflexion point [1], whereby advances in computational methods, wearable sensing technology and open access to Big Data are reshaping the ways in which we inform basic science and rapidly translate our knowledge to actionable treatments. Psychiatry is one of those medical fields that is rapidly evolving, while adapting traditional models to help advance the main goal of helping patients improve their quality of life. Along those lines, computational psychiatry [2], a nascent subfield within psychiatry, is merging methods from Computational Neuroscience with clinical approaches through successful collaborations. These new developments are bound to open new frontiers in therapeutic treatments. Further, as part of a more general effort in the medical field, precision medicine (PM) [1] has emerged as a new platform to combine expertise from multiple layers of the knowledge network in order to ultimately design personalized targeted treatments (Figure 1A). Integrating the personalized concept of PM with the new advances in computational psychiatry could give us a new way to approach mental illness and help patients cope with lifelong changing needs.

The task ahead is challenging because there is no proper roadmap to connect the layers of the knowledge network in PM and produce personalized diagnoses and measures of treatment outcomes that truly separate disease progression from treatment effectiveness according to age and development. Part of the problem is that most brain science has focused on experimental assays and methods that curtail natural movements. As such, our knowledge about the dynamics of natural behaviors is very limited, particularly in reference to those aspects of behavior that remain hidden to the naked eye of the clinician trained to observe specific expected behavioral landmarks of a psychiatric disorder conceived exclusively as a mental illness. In so doing, the clinically trained eye may miss important information that is perhaps common across different disorders of the nervous systems and rather relevant to help improve the patient’s quality of life. For example, motor coordination and volitional control are critical ingredients of autonomy in any natural behavior underlying activities of daily living. Yet, these are not considered part of the diagnostics criteria for mental illnesses such as autism, schizophrenia and depression, as per the Diagnostics Statistical Manual (DSM-5) [3] (and see Supplementary Materials).

Research on the underlying neurobiology of mental illnesses has revealed their associated genetics [4] and/or helped characterize patterns of brain activity in response to external stimuli [5] (while curtailing naturalistic bodily motions to avoid instrumentation artifacts in imaging data or in EEG, MEG, etc.). This central approach to brain science has left us with a paucity of information about the possible contributions to mental illness from the peripheral nervous systems, and from vital organs important for autonomous living. The peripheral activity, however, continuously feeds back to the brain via afferent (body-to-brain) channels and is, in turn, dynamically updated through efferent (brain-to-body) activity, self-generated by the system itself. This recursive loop, whereby re-entrant information that is partly self-produced by the organism and partly influenced by external environmental conditions, would provide important clues about truly evolving dynamics and stochastic (variability) across all-natural behaviors. Approaching the problem through this lens could bring a new quantifiable layer of granularity to basic research. This would include the design of age-appropriate metrics reflecting the development of the organism as it ages and as it copes with a disorder [6,7]. The micro- and macro-motion data from the nervous systems biorhythms is the low hanging fruit that we can easily attain by leveraging the wearable sensors revolution. Further, because these quantifiable digitized activities and signals therein are partly self-generated, self-monitored and self-corrected by and within the nervous systems, this quantitative approach has the potential to take us from a purely correlational science to a science that is based on causal relations between nervous system activities and external/contextual stimuli. In this sense, the new proposed approach to mental illness is amenable to intervene and modify the system with well-informed, near-optimal means capable of improving its performance.

Micro- and macro-motions that underly all aspects of human behavior depend on the intactness of fundamental tissues, many of which have already been characterized in genomics according to cell types [8]. Here, we propose to combine micro-level underlying aspects of behavior with the current genomics knowledge to inform psychiatry of possible ways to improve quantification of nervous system activities. Ultimately, we seek to compile this information to help build accommodations and support for the patient population, while reconceptualizing mental illness as a physically quantifiable disorder of *the nervous systems*.

The nervous systems already offer a taxonomy of function and control that is phylogenetically ordered and well organized along several axes. Some of these axes are accessible today with non-invasive means and, as such, we can obtain signals and build computational models to understand mechanisms and translate them to actionable societal solutions. One possible orderly structure is suggested in Figure 1B, where we propose to map levels of neuromotor control (voluntary, involuntary, and autonomic) to fundamental types of muscles (skeletal, smooth, and cardiac) linked to commonly sampled tissues in genomic datasets. Combining information about gene expression on tissues that involve key components of the central nervous systems (the brain and the spinal cord), key organs for vital bodily functions (including smooth muscle lining internal organs), muscle-skeletal tissues and nerves, and cardiac tissues (for autonomic heart functioning), we explore the effects of removing disease-associated genes, on the overall remaining genome expression on these tissues. As a first step in this exercise, we reasoned that the genes associated with a given disorder ought to be important in the functioning of certain systems, which in turn depend on certain tissues. We also reasoned that such stochastic variations and combinations could be measured relative to the presence of all genes and to the absence of genes across neurological or non-neurological conditions.

What is the tissue distribution of gene expression in neuropsychiatric disorders such as autism, schizophrenia, and depression in relation to well-characterized neurological conditions? Is there convergence in the remaining gene expression on the tissues upon removal of the genes associated with that disease? Furthermore, how would the gene expression change across the tissues in non-neurological conditions such as various forms of cancer, immunodeficiencies, endocrine system deficiencies and so forth? How would it change in acquired disorders such as Post Traumatic Stress Syndrome (PTSD), currently diagnosed through observation?

Take autism for example. Autism is an umbrella term for a very heterogeneous set of neurodevelopmental disorders, but no gold-standard criteria include core neurological symptoms that could help us create early accommodations and support for the nascent nervous systems of the infant (during pre-cognitive stages of neurodevelopment). The rule of thumb is to assume that the child has odd, socially inappropriate behaviors and that they should be modified through operant and cognitive conditioning techniques—often translated from lab animals to human babies, without any type of collaboration with other fields studying infant development. Current methods of diagnoses and treatments in autism are not based on normative neurodevelopmental data charts to understand age-dependent departures from typical neurodevelopment. Without any systematic way to build age-appropriate metrics in order to capture highly non-linear, stochastic patterns and rates of change in the (rather accelerated) infant neurodevelopment, entire generations of infants, children and adolescents have been exposed to such means of behavioral treatments and no information can tie these back to the underlying genomic pool of this population.

In schizophrenia, delusions, avolition and catatonia are at the core of the disorder, but as in autism above, no criteria in the DSM highlight the profound somatic sensory-motor issues that have been found in patients [9]—even without the use of psychotropic medications known to alter motility. Interestingly, historical accounts of psychiatry (in pre-Freudian times) show the reliance on motor aspects of the behaviors that defined several mental illness from a neurological perspective [10].

Depression is also currently treated purely as a mental illness, but it may be important to understand potential contributions to various forms of depression, from the peripheral nervous systems and from the body in general. Genetic information may give us a way to link tissues affected in these neuropsychiatric conditions with those affected in neurological conditions, for which treatments and interventions of various forms may be effective. These may be in the form of drugs, or in the form of physical, mindfulness and occupational therapies aimed at helping support the person’s bodily autonomy and overall increase the chances for independent living.

We here offer a new lens to help balance psychiatric with neurological criteria derived from genomic information specific to each disorder. In a first (crude) step of many to come, we start by comparing well-known neuropsychiatric and neurological conditions, the results from eliminating the genes associated with each disorder and quantifying the degree of convergence in the maximally affected tissues, in relation to those resulting from eliminating the genes associated with non-neurological conditions. We focus our discussion on possible ways to continue this path of inquiry and highlight current caveats for future improved iterations of the proposed methods.

## 2. Materials and Methods

We combine the datasets from genes associated with mental illnesses with well-known neurological disorders and with illnesses that are not directly associated with the nervous systems. We also include genes associated with manifestations of acquired Post-Traumatic Stress Syndrome Disorder (PTSD). Among mental illnesses defined by the DSM-5, we include autism, schizophrenia and mental depression of different types, (e.g., general, bipolar and unipolar). Among neurological conditions, we include ataxias (e.g., cerebellar, spinocerebellar, progressive, and gait) and Parkinson’s disease. Among non-neurological disorders, we include colon cancer, breast cancer, diabetes, congenital heart disease, hematologic neoplasm, and various autoimmune disorders (lupus systemic erythematosus, psoriasis, and irritable bowel syndrome).

We use the genes, gene expression, and tissues from the Genotype-Tissue Expression project, GTEx pPortal human RNA-seq (Transcripts Per Million (TPM), see Appendix A for note in TPM) as reference specifically using the files denoted in Appendix B. In autism, we use the gene scoring module of the Simons Foundation Autism Research Initiative (SFARI) scored according to evidence from the literature. We also use ataxia genes, the X genes and the FX genes taken from various literature reviews [11,12]. Furthermore, we use genes associated with mitochondrial disorders [13] and genes identified in Parkinson’s disease, taken from [14,15,16,17,18,19]. Besides the autism SFARI genes and the genes reported in literature reviews, we take the genes associated with autism, schizophrenia and depression reported in https://www.disgenet.org/home/ along with other genes from the above-mentioned non-neurological disorders. The latter will inform us of fundamental differences in gene expression between these diseases and those which affect neuromotor control and basic functioning, as mediated by interactions between the brain and the peripheral nervous systems (including the autonomic nervous system).

The SFARI autism categories that we used were those reported as of 03-04-2020. Quoting from their site:

- CATEGORY 1
  Genes in this category are all found on the SPARK gene list. Each of these genes has been clearly implicated in Autism Spectrum Disorders, ASD—typically by the presence of at least three de novo likely-gene-disrupting mutations being reported in the literature—and such mutations identified in the sequencing of the SPARK cohort are typically returned to the participants. Some of these genes meet the most rigorous threshold of genome-wide significance; all at least meet a threshold false discovery rate of <0.1.
- CATEGORY 2
  Genes with two reported de novo likely-gene-disrupting mutations.
  A gene uniquely implicated by a genome-wide association study, either reaching genome-wide significance or, if not, consistently replicated and accompanied by evidence that the risk variant has a functional effect.
- CATEGORY 3
  Genes with a single reported de novo likely-gene-disrupting mutation.
  Evidence from a significant but unreplicated association study, or a series of rare inherited mutations for which there is not a rigorous statistical comparison with controls.
- SYNDROMIC
  The syndromic category includes mutations that are associated with a substantial degree of increased risk and consistently linked to additional characteristics not required for an ASD diagnosis. If there is independent evidence implicating a gene in idiopathic ASD, it will be listed as “#S” (e.g., 2S, 3S). If there is no such independent evidence, the gene will be listed simply as “S”.

The GTEx dataset is as the 06-05-2017 v8 release. For every gene in autism, ataxia, X, FX, mitochondrial diseases, Parkinson’s disease, and the non-neurological diseases, we first confirmed the presence of the gene in the GTEx dataset and then incorporated it into the analyses.

The genes from the DisGeNet portal were found by interrogation of their dataset under disease type and saving the outcome to excel files containing all pertinent information. All sample files used in our analyses are provided in Supplementary Materials.

### 2.1. Count Normalization

The GTEx matrix of RNA-seq genes along the rows (56,146) × the tissues (54) along the columns was transposed (54 × 56,246), such that we expressed each tissue as a function of the gene expression denoted by the count (TPM). Each individual count value was then normalized using Equation (1).

(1) $$Normalized\;Count=\frac{count_{i}}{count_{i}+\frac{Avrg_{Global}Count}{Max_{Global}Count}}$$

**Figure 2:**
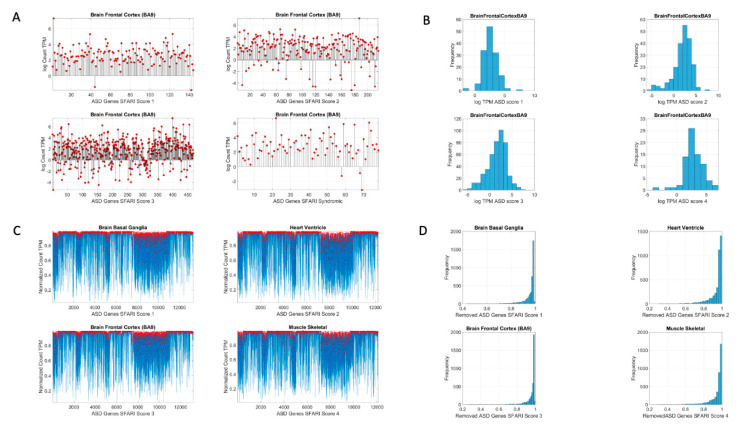
Analytical methods. (**A**) Sample raw data consisting of the log count (TPM) for different scored genes expressed in the brain frontal cortex (Brodmann Area 9). (**B**) Histograms of the log count TPM for each case in (**A**). (**C**) Upon removal of the Simons Foundation Autism Research Initiative (SFARI) autism genes, we obtain micro-fluctuation spikes in the normalized count, with deviations taken relative to empirically estimated mean, global averaged count, and global maximal count in Equation (1). (**D**) Histograms of the normalized micro-fluctuation spikes.

Here, *count_i_* is the count value of the gene_i_, *Avrg_Global_Count* is the overall average of the matrix of values taken along the columns and the rows. *Max_Global_Count* is the maximum count value, also taken globally across the matrix values. Figure 2 shows the original count numbers (Figure 2A) and the normalized version (coined micro-movement spikes (MMS)) in Figure 2B. Figure 2C shows the MMS derived from the fluctuations in counts normalized by Equation (1), while Figure 2D shows the histograms of the peaks (marked in red dots) for different tissues and genes scored by the SFARI.

### 2.2. Gene Removal

For each of the disorders of interest in Appendix B Table A1 (mental illness and neurological) and Table A2 (non-neurological), we remove the genes associated with each condition from the human GTEx dataset. These disease-gene associations are as reported in the various databases (the SFARI, DisGeNet https://www.disgenet.org/home/ and the literature meta reviews). We then treat the resulting count series as a random process. We use the exponential distribution to characterize it and to assess the differential expression across the tissues relative to non-removal in the original human genome.

The question that we ask is: given the known neurological phenotypes, is there convergence between the most-affected tissues upon associated gene removal and the changes in the tissues that will be obtained by the removal of genes associated with mental illnesses? Furthermore, is there convergence with the outcome from removing the genes associated with non-neurological illnesses? Appendix B Table A1 and Table A2 show the number of genes removed in each respective case as well as the source of the reported genes associated with each condition/disease.

### 2.3. Stochastic Analyses

Since the count values for each tissue can be conceived as a random series of numbers, we use maximum likelihood estimation (MLE) to model the numbers representing the counts, as generated by the exponential distribution using Equation (2)

(2) $$y=\lambda e^{-\lambda x}$$

**Figure 3:**
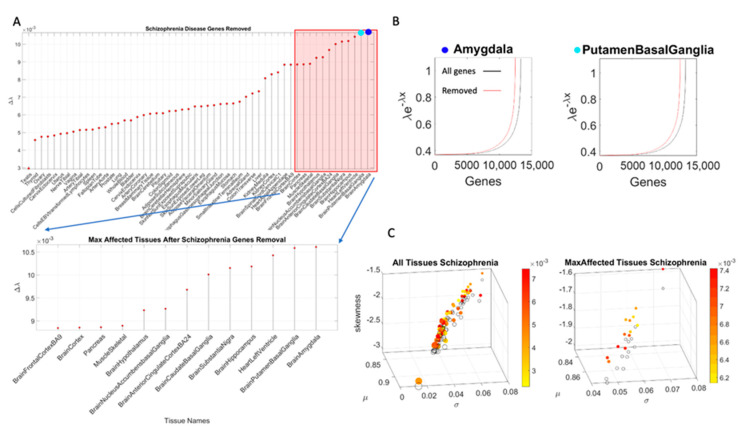
Sample metrics used for the stochastic analyses of a data sample (using 2697 schizophrenia-associated genes reported in DisGenNet portal and in the literature). (**A**) Effect of removing the schizophrenia genes from the GTEx human genome set expressed across 54 tissues. Tissues are sorted in ascending order, by the absolute difference Δλ between gene expression on the 54 tissues before and after removal. The red square highlights the top 13 median-ranked tissues shown in the panel below and the dark and light blue circles mark the top two tissues affected (the brain amygdala and brain putamen in the basal ganglia. (**B**) The exponential distribution curve is fit to the sorted normalized count representing the gene’s expression in TMP on the top median-ranked affected tissues (as in Figure 2C, taking the peaks highlighted in red and fitting the exponential distribution to the frequency histogram, as in Figure 2D) before (black line) and after (red line) the removal of the genes associated with the disease. The absolute value difference between the curves is the Δλ used to rank the tissues by the effect size. (**C**) The fitting of the gamma distribution yields the shape and scale parameters used to compute the gamma moments. The axes represent the mean, the variance, and the skewness of the distribution of the normalized values and the color map represents the Earth Mover’s Distance values measuring the difference between the resulting exponential frequency histograms in (**B**). The size of the circle is proportional to the kurtosis and the color-filled circles represent the tissue (54 in the left panel) with the original gene expression from GTEx (our reference template) vs. the open circles representing the stochastic shift, i.e., upon the removal of the genes associated with the disease in DisGenNet. The right panel contains the top-ranked tissues (13) according to the median values of the Δλ.

Here, *x* represents the normalized count value (as per Equation (1)) and *y* represents the value from the exponential distribution. We seek the value of the rate parameter *λ* to model this random counting process, which we use to represent the gene expression in the tissue. To that end, we estimate the likelihood $L\left(\lambda |x_{1},x_{2},\ldots ,x_{n}\right)$ where the series of counts *x_i_*, with *i* ranging from 1 to *n*, represent the normalized counts (according to Equation (1)) across all genes for one tissue. Appendix B shows the steps to find *λ*. Further, this is computed for each of the 54 tissues. We then rank the departure of *λ* (resulting from gene removal) from the *λ* obtained for the full human genome (see below). This is explained in Figure 3.

### 2.4. Stochastic Analyses—Visualization of Change Relative to the Normative Data of the Full Human Genome

Using MLE, we also obtain for each of the 54 tissues, the frequency histograms of the normalized counts across all genes and fit the continuous gamma family of probability distributions with shape (*a*) and scale (*b*) values in order to obtain the gamma moments and plot them on a parameter space. We do this to visualize the spread of the tissues and their shift upon gene removal. To that end, we plot the mean, the variance, and the skewness across the *x*, *y* and *z* axes, respectively. We plot the size of the marker representing the tissue proportional to the kurtosis value, and we color the marker based on the change relative to the original genome count (i.e., containing all the genes, without removal).

To measure the stochastic shift between the tissues from the full genome and those upon removal of the genes identified with each known neurological condition, we take the absolute difference between the MLE λ for the full GTEx genome and that for the genome upon removal of the genes associated with each condition, disorder or disease, as shown in Figure 3.

We median rank the Δλ for each tissue, sorting Δλ in ascending order across the 54 tissues. Then we create four median-ranked blocks and plot the maximally affected block of tissues (Figure 3A). The highest-ranked group is then compared across all conditions—mental illness vs. those neurologically defined vs. those non-neurologically defined. We annotate the neurological functions that such tissues are known to maximally disrupt. Further, we determine whether there is convergence between the tissue outcome in mental illnesses, upon removing the associated genes from the human genome-tissue model, and the outcome upon removing those genes tied to the other known disorders of the nervous systems. We repeat this interrogation process using the genes associated with disorders in the DisGeNet portal, including autism, schizophrenia, depression, the neurological disorders in Appendix B Table A1, and the non-neurological disorders in Appendix B Table A2.

To assess tissue outcome upon removal of genes associated with various non-neurological diseases, we follow these procedures and compare these to the above results. These diseases include colon cancer, breast cancer, psoriasis, diabetes, congenital heart disease, hematologic neoplast and systemic lupus. Appendix B Table A2 describes the number of genes associated with each of these diseases and the sources.

We also examine other mental illnesses described by the DSM. These include schizophrenia, depression, unipolar depression, and bipolar depression. We determine whether there are tissues that overlap with those affected in the neurological disorders. Lastly, we examine mitochondria-related disorders and PTSD using these methods. We reasoned that these may be disorders that have potentially affected tissues across a broader range of functions, including those from the brain and other bodily organs.

## 3. Results

### 3.1. Autism, Ataxia and FX Have Convergence in Maximally Affected Tissues by the Removal of Associated Genes

**Figure 4:**
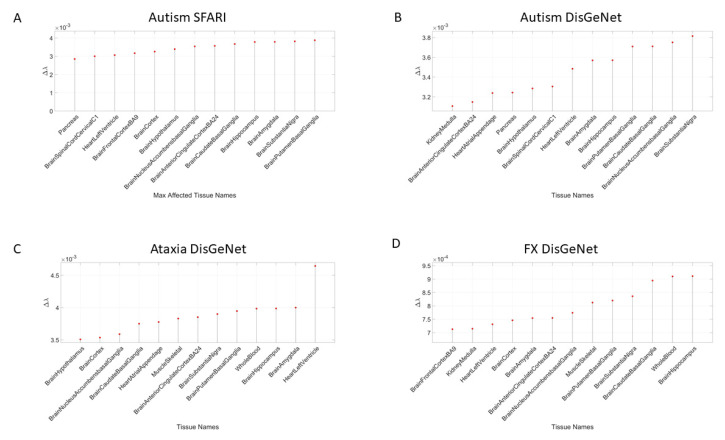
Convergence between autism and known neurological disorders shown by comparison of maximally affected tissues after removing genes associated with the disorder in autism (from the SFARI), autistic disorders (**A**,**B**) (from the DisGeNet portal) vs. ataxia (**C**) and fragile X (**D**) (See Appendix B Table A3 and Table A4).

The maximally affected tissues upon gene removal, according genes stochastic expression (count in Transcripts Per Million (TPM)), are depicted in Appendix B Table A3. Tissue gene expression was modelled by the exponential distribution $y=\lambda e^{-\lambda x}$, with *x* as the gene combination expressed in the tissues, and λ as the exponential rate parameter. The Δλ between the neurotypical template case from the GTEx portal (containing all genes) and the modeled disorder case (upon removal of the SFARI genes) provides a sense of the departure from the normative case. This difference, taken for the removal of the SFARI genes, is depicted in Figure 4A, with samples of maximally affected tissues in Figure 4B that are known to be critical for motor control, regulation, adaptation/learning, and coordination.

The Δλ median ranking quantified the difference between neurotypical tissue gene expression vs. tissue gene expression upon removal of the genes corresponding to the disorders in question, with four groups ordered by the size of Δλ. This λ quantity was first obtained relative to the neurotypical population tissues, i.e., including all the counts (gene expression) from all genes, in order to model an exponential process. The computation of λ using maximum likelihood estimation (MLE) is explained in Appendix A. It does not assume any order of the counts, but rather seeks to identify the resulting λ for each tissue, treating the gene count (expression) as a random, memoryless stochastic process. Typically, the exponential distribution is used to model times between events, but here we used it to model the fluctuations in the values of the counts across the genes, as they randomly fluctuate their expression across each of the 54 tissues reported in the GTEx portal. We note this to underscore that the results spontaneously self-emerge from the random combination of the genes involved (with and without removal), rather than from the clinical criteria used to denote gene relevance to autism, or the evidence from the literature used to determine their association. There is in fact no scoring of such relevance for the genes associated with the other neurological disorders under consideration (e.g., ataxias and Parkinson’s disease) or for the autistic disorders reported in the DisGenNet portal. Using those genes instead of the SFARI genes reveals tissues in Appendix B Table A4, where we report the convergence.

Removal of the SFARI genes, ranked by change in gene expression, reveals brain tissues linked to the CNS, in brain subcortical tissues linked to motor control (basal ganglia, striatum), memory (hypocampus), emotions (amygdala) and regulation (hypothalamus); and the spinal cord. This is also generally the case for the removal of the DisGeNet genes associated with autistic disorders and with FX and ataxia. Congruent with the outcome from the removal of the SFARI genes from the GTEx genome, the DisGeNet gene removal also affected the tissues associated with CNS function. Important tissues for systemic organ functioning such as those containing smooth muscles, cardiac and skeletal muscles in the taxonomy proposed in Figure 1B were also affected (commonly) across these disorders. Figure 4 shows a summary of the results, visualizing the stochastic shifts. Appendix B Table A3 and Table A4 show the tissues ranked in descending order and color coded according to CNS (brain and spinal cord in blue), heart related (pink), muscle-skeletal (green), and peripheral vital organs (gray). Most tissues in autism and the neurological disorders are from the CNS, followed by PNS-related tissues in the heart and muscle-skeletal and with vital organs towards the end of the Δλ ranking.

Supplementary Figures S1–S3 show these results separately for each neurological condition. We note in Supplementary Materials that removal of the SFARI autism syndromic genes from the GTEx genome reveals maximal differences in tissues of organs with smooth and cardiac muscles linked to involuntary and autonomic function in the proposed taxonomy of Figure 1B.

**Figure 5:**
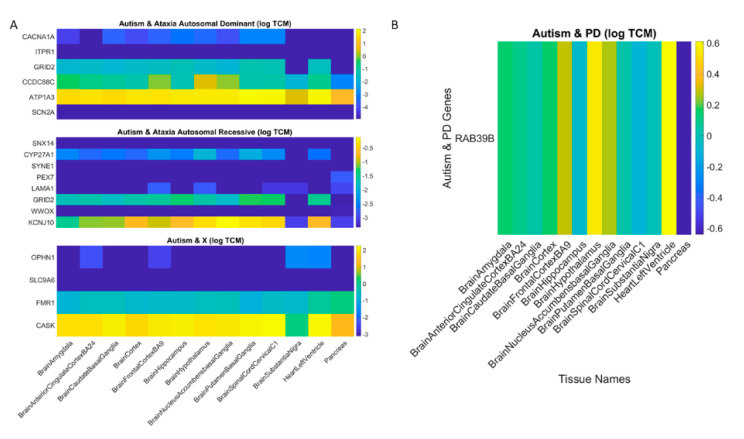
Gene expression on maximally affected tissues (color bar coded in log TPM) upon removal of overlapping genes between the SFARI autism set and ataxias (dominant and recessive and X-chromosome sets) from the literature (**A**) and (**B**) from Parkinson’s disease. The horizontal axis lists the tissue names and the vertical axis lists the gene names.

Removal of the overlapping SFARI genes and neurological disorders also reveal brain tissues linked to motor control, memory, emotions, and regulation. This is depicted in Figure 4. Given the congruence between the tissues maximally affected by removing the SFARI autism genes from the GTEx database and those from the neurological conditions, we next ascertain the extent to which these genes overlap with those used from ataxias in the literature. To that end, we divide them into the autosomal dominant, the autosomal recessive and the X-chromosome genes. Figure 5A shows the result upon removal of overlapping genes between the SFARI autism set and ataxias (dominant and recessive and X-chromosome sets) from the literature. Appendix B Table A3, Column 3 lists the tissues, while Appendix B Table A5 also has the scoring from the SFARI autism genes. Supplementary Materials Table S2 lists the phenotypic information of the disorders associated with these genes, as described by the clinical literature. Figure 5B lists the PD gene that overlaps with the SFARI autism genes, also depicted in Appendix B Table A5 along with the score ranking from the SFARI portal.

We note that removing this subset of 14 overlapping genes from the SFARI autism set (Appendix B Table A5) does not change the primary result, whereby the most-affected tissues upon removal of the SFARI autism set from the GTEx dataset are those associated with subcortical brain structures critical for motor control, adaptation/learning, regulation, coordination and autonomic function as well as memory and emotion. This is shown in Figure 5A,B and in the third column of Appendix B Table A3. In Figure 4D, we also plot the top-ranked tissues affected by the removal of the FX genes reported in the DisGeNet portal from the GTEx dataset. Supplementary Figures S19–S22 further provide details on X-chromosome genes in Figure 5A implicated in autism according to the SFARI genes portal.

The result that convergence in the ranked descending order of the CNS (brain and spinal cord tissues), followed by heart-related tissues, muscle-skeletal tissue and lastly peripheral vital organs for systemic functioning in the SFARI autism and well-known neurological disorders from the literature is also congruent with the results using the genes associated with these conditions in the DisGeNet portal. There, we interrogated autistic disorders, ataxias and fragile X, confirming the overlap in genes, their expression on the 54 tissues of the GTEx database and the orderly levels of tissues maximally affected by the removal of the associated genes. We grouped the tissues by CNS, heart, muscle-skeletal and peripheral vital organs to follow the proposed taxonomy of Figure 1B.

**Figure 6:**
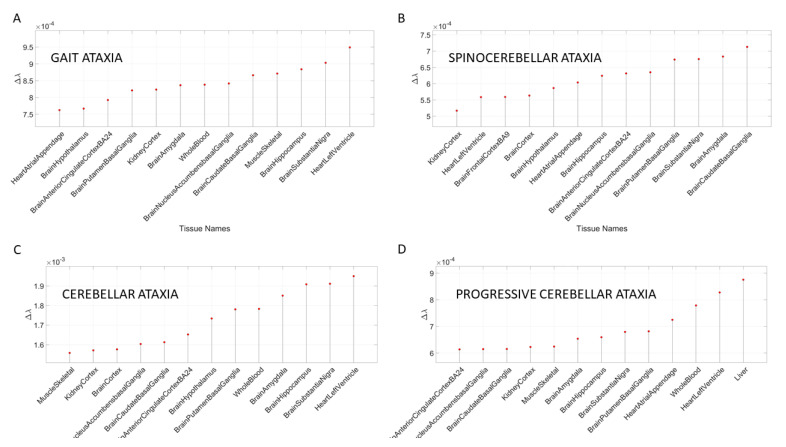
Different types of ataxia showing the top Δλ-ranked tissues, also shown in Appendix B Table A7. (**A**) Gait ataxia; (**B**) spinocerebellar ataxia; (**C**) cerebellar ataxia; (**D**) progressive cerebellar ataxia.

In the remaining sections of this paper, we consistently use this tissue grouping to simplify visualization of the Appendix B tables and data presentation. Figure 6 shows the results for different types of ataxias and FX, while Appendix B Table A6 summarizes the top-ranked affected tissues in different types of ataxias. These are color coded according to the tissue grouping, approximating the taxonomy proposed in Figure 1B.

### 3.2. Removal of Genes Associated with Schizophrenia and Multiple Forms of Mental Depression Reveals Convergence with Neurological Disorders

**Figure 7:**
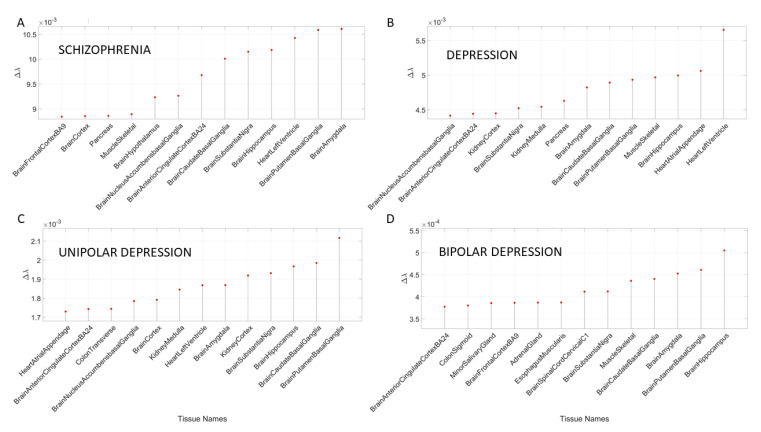
Maximally affected tissues in schizophrenia (**A**) and depression (**B**), and in different types of depression (unipolar (**C**) and bipolar (**D**)) shown in Appendix B Table A7.

The removal of the DisGeNet genes associated with mental illnesses such as schizophrenia, depression, bipolar depression and unipolar depression from the normative GTEx genome resulted in convergence of maximally affected tissues involved in the CNS, especially those brain regions necessary for neuromotor control, memory, and emotion. This is depicted in Appendix B Table A7 and Figure 7. Several of these tissues were also found to be affected upon removal of the SFARI genes and the genes associated in DisGeNet with autistic disorders. Furthermore, these are maximally affected tissues in the well-known neurological conditions depicted in Figure 4 and Figure 5 and Appendix B Table A3, Table A4 and Table A5.

### 3.3. Removal of Genes Associated with Non-Neurological Disorders Reveals Other Non-CNS Tissues

**Figure 8:**
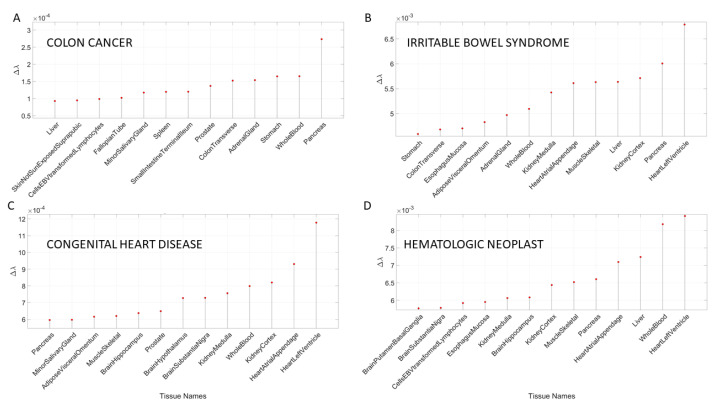
Maximally affected tissues in a sample non-neurological diseases of Appendix B Table A8 reveal primarily non-CNS tissues involving peripheral vital organs for systemic functioning, followed by heart-related and muscle-skeletal tissues. As before, the interrogation of the GTEx genome is based on the genes associated with diseases in the DisGeNet portal. (**A**) Colon cancer; (**B**) irritable bowel syndrome; (**C**) congenital heart disease and (**D**) hematologic neoplast. Color code as in previous tables.

**Figure 9:**
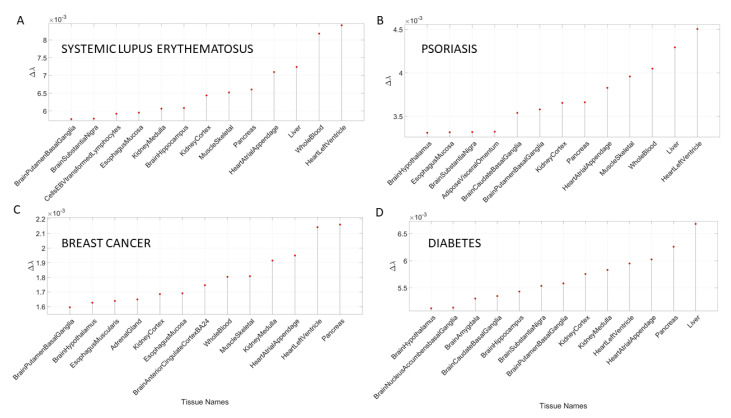
Maximally affected tissues in non-neurological diseases. (**A**) Systemic Lupus Erythematosus; (**B**) Psoriasis; (**C**) Breast Cancer; (**D**) Diabetes. These are depicted in Appendix B Table A10 median-ranked according to the Δλ values obtained from the absolute difference between the tissues according to the full genome in the GTEx database and the GTEx genome without the genes associated with each disease, according to the queries to the DisGeNet portal.

In addition to the examination of mental illnesses and neurological disorders, we also interrogated the GTEx genome upon removal of genes associated with various non-neurological disorders. These included various forms of cancers, inflammatory and autoimmune disorders and other tissues related to the heart, the circulatory and the endocrine systems. Appendix B Table A8 and Table A9 summarize the results of this interrogation and Figure 8 and Figure 9 show the Δλ-ranking graphs.

The results of the maximally affected tissues upon the removal of the genes associated with these non-neurological disorders revealed a very different picture than those upon removal of the genes associated with the mental illnesses (autism, schizophrenia and the depressions) and those associated with the known neurological conditions (the various forms of ataxia, FX and Parkinson’s disease). Namely, the CNS-related tissues were less affected in these non-neurological diseases than those related to the PNS (muscle-skeletal and ANS heart), and those linked to peripheral bodily organs were the most visibly affected. The exception was diabetes, maximally affected tissues in peripheral organs, but also CNS and PNS tissues in the tail of the top Δλ-ranked tissues. We next interrogate the genome in relation to mitochondrial disorders of several kinds and acquired PTSD.

### 3.4. Removal of Genes Associated with Mitochondrial Diseases Reveals that Heart-Related Tissues Are Maximally Affected but PTSD Is Mixed

Removal of genes associated with mitochondrial disorders of various types from the GTEx genome, according to the genes in the DisGeNet portal, reveal a mixture of tissues associated with peripheral vital organs for systemic functions, heart-related and muscle-skeletal- and CNS-related tissues. The top half of the highest-ranked tissues in mitochondrial disease shows affected tissues related to the heart, muscle-skeletal and peripheral organs, while the bottom half shows more involvement of brain-related tissues in subcortical regions of motor control. In contrast, mitochondrial myopathies show a predominance of CNS-related tissues, including the brain and spinal cord, with top Δλ-ranked tissues related to the heart and muscle-skeletal tissues. Mitochondrial encephalopathy, lactic acidosis, and stroke-like episodes (MELAS) show a predominance of tissues associated with peripheral vital organs for systemic function and heart-related tissues. Only two brain regions for motor control and emotion are present in the bottom-ranked tissues of the most-affected tissues.

**Figure 10:**
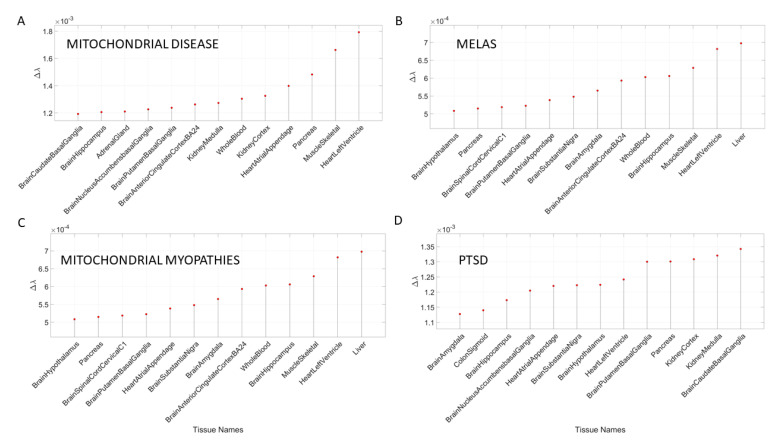
Maximally affected tissues in mitochondrial diseases (**A**–**C**) and in PTSD (**D**) of Appendix B Table A10, shown in graphical form according to the Δλ values.

The case of acquired PTSD also reveals a mixture of tissues from brain, heart, and peripheral organs. There, we see maximally affected tissues linked to subcortical regions of the brain involved in motor control, adaptation, learning, and coordination intermixed with tissues linked to peripheral bodily organs (like the kidneys) and the autonomic systems’ heart. Furthermore, we also see tissues linked to the hypothalamus, a regulatory brain structure. Figure 10 shows the graphs of the Δλ difference, which was median ranked, as in the previous cases, for these disorders.

**Figure 11:**
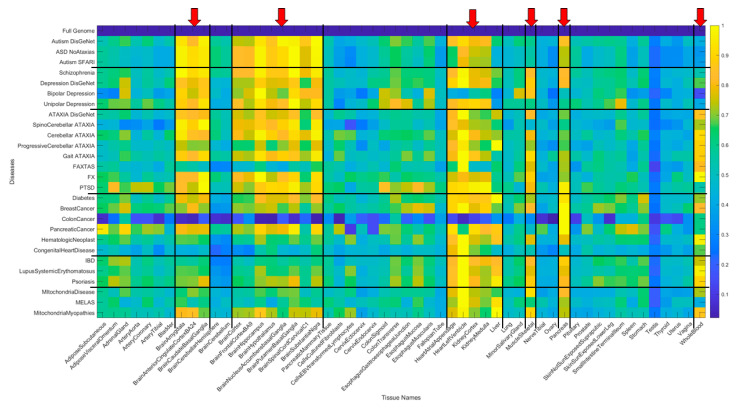
Summary of disorders/diseases (27 rows) x tissues (54 columns) in alphabetical order. Entries are Δλ (difference with respect to the gene expression values from the full GTEx genome) values normalized by the maximum across the tissues for each row (disorder/disease). The first row is the 0–Δλ difference reference from the full genome. Red arrows mark the maximally affected tissues across all diseases, showing mental illnesses on the top, followed by neurological disorders, then non-neurological, including several types of cancer, autoimmune disorders, and diabetes. Black lines delineate blocks of diseases (along rows) and blocks of gene expression on tissues (along columns).

We summarize the results across all 54 tissues (in alphabetical order from left to right) in Figure 11. Here, a color map depicts the values of Δλ normalized for each disease (along the rows) across the tissues (columns) by dividing by the maximum Δλ value of each row. The patterns reveal that the maximally affected tissues (upon genes removal) are common to both neurological disorders and mental illnesses. They correspond to the brain tissues involved in motor control, adaptation, and learning (basal ganglia, striatum, substantia nigra), tissues in involved in emotion (amygdala), memory (hippocampus) and systemic regulation (hypothalamus). They also reveal that whole blood tissue is not as affected in the mental illnesses as in the neurological disorders (marking a point of divergence that warrants further investigation). Heart-related tissues and muscle-skeletal tissues are also shared between these mental illnesses and neurological disorders when the genes specific to each disorder are removed from the GTEx genome. Interestingly, the pancreas is an example of a peripheral bodily organ with tissues that are commonly affected across most of the disorders and diseases interrogated here. Yet they have lower weight the neurological disorders compared to the non-neurological diseases under examination.

**Figure 12:**
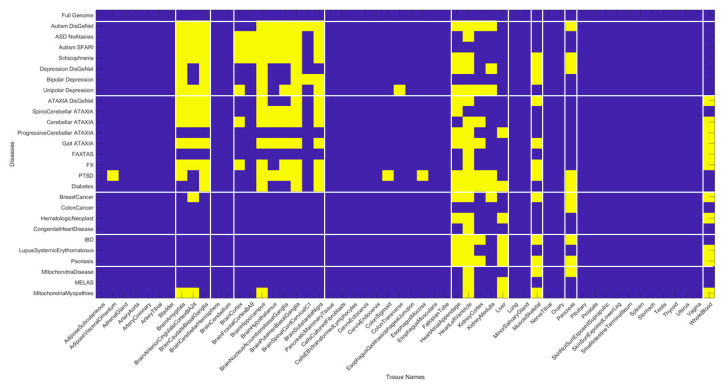
Binary version of the matrix in Figure 11 upon thresholding by a high normalized Δλ value of 0.8 shows that the overlap across mental illnesses, neurological disorders and non-neurological diseases is primarily in the heart tissues, the muscle-skeletal tissues and organs such as the pancreas, liver, and kidney. Whole blood tissue is shared between neurological and non-neurological disorders but not present in the mental illnesses. Brain tissues are shared between mental illnesses and neurological disorders.

The non-neurological diseases reveal less involvement of the CNS-related tissues but highly overlap with the heart and muscle-skeletal tissues. Tissues linked to the kidneys, liver and pancreas are also maximally affected by genes’ removal in these diseases. Colon cancer shows an interesting pattern whereby the pancreas reveals maximal normalized Δλ values. Figure 12 summarizes the patterns in binary form by turning ON (yellow) values above 0.8 considered high and OFF those below (blue). This cut off is chosen to further highlight overlap and differences across diseases based on high Δλ.

**Figure 13:**
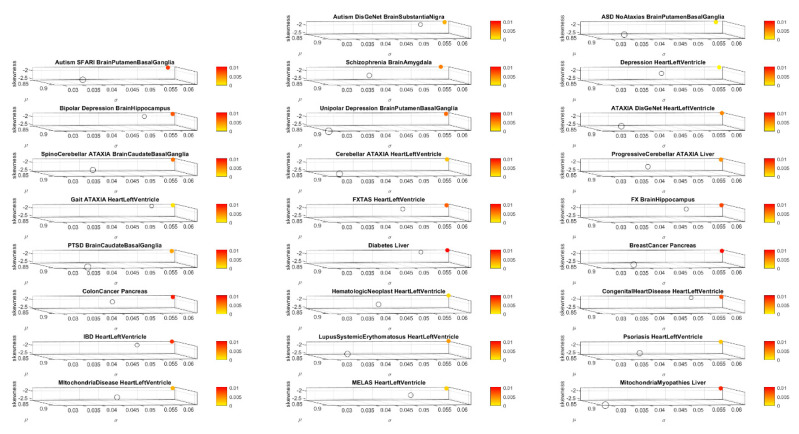
Stochastic shift of gene expression on tissue after gene removal for each disease/disorder under consideration (colored circle) relative to the gene expression on that tissue using the full genome in GTEx (open circles). The most-affected tissue in each disorder/disease under consideration was selected according to the maximum Δλ value, the absolute difference between the empirically estimated λ using MLE of the exponential distribution rate parameter for the full GTEx genome and for the genome minus the genes associated with each disease/disorder. Since the exponential distribution is a particular case of the continuous gamma family of probability distributions (when the gamma shape parameter is 1), we also used MLE to estimate the shape and scale gamma parameters and the four gamma moments, plotted here in a five-dimensional parameter space. Along the *x* axis, we plot the empirically estimated gamma mean; along the *y* axis, we plot the gamma variance; along the *z* axis, we plot the gamma skewness and the kurtosis is used to represent the size of the marker (more kurtotic distributions have higher value, i.e., larger circle). The fifth dimension is the color representing the Δλ (see also Figure 3C, visualizing one single disease and all 54 tissues or summarizing the top 13 median-ranked tissues as those most affected).

The patterns revealed by the high values of the normalized Δλ quantity, show convergence of maximally affected brain tissues in mental illnesses with the neurological disorders but not with the non-neurological disorders (except for diabetes which does affect some brain regions.) The mitochondrial diseases do not show the same intensity of the CNS-related Δλ values as the mental illnesses and the neurological disorders, but they do share the heart and muscle-skeletal patterns with all the examined diseases and disorders. This is interesting, given that some of the children with various forms of mitochondrial disorders may receive diagnoses of autism. In summary, there is clear overlap between mental illness and neurological disorders, suggesting involvement of the central nervous systems in both. We also see major contributions from the peripheral nervous systems, particularly the heart, the muscle-skeletal tissues and, to a lesser degree, tissues of peripheral organs. The latter are most affected in the non-neurological diseases. Figure 13 shows the most-affected tissue in each disease/disorder (also depicted in Appendix B Table A11).

## 4. Discussion

This work interrogated the human genome by removing genes associated with various diseases and comparing the outcome from the remaining gene expression on 54 tissues commonly examined in the GTEx portal. These tissues involve parts of the central nervous systems (the brain and the spinal cord) and parts of the peripheral nervous systems (the muscle-skeletal tissue and the heart tissues as part of the autonomic nervous system), which we grouped in Figure 1B. Other tissues are from peripheral vital organs for systemic bodily functions (whole blood, pancreas, liver, kidneys, lungs, etc.).

We compared mental illnesses such as autism, schizophrenia, and various types of mental depression (including unipolar and bipolar), with well-known neurological disorders such as different types of ataxias, fragile X and Parkinson’s disease. We found convergence between the tissues maximally affected by the removal of disease-associated genes across these mental illnesses and neurological disorders. CNS-related tissues in subcortical regions of the brain related to motor control, motor learning, motor coordination, and motor adaptation, as well as memory and emotion, were predominantly maximally affected by the corresponding gene removal across both the mental illnesses and the neurological disorders. This convergence demonstrates overlap between psychiatric and neurological conditions with specific involvement of motor, memory, emotional and regulatory axes. In autism and FX, we obtained congruent results on maximally affected tissues. The results were consistent using removal of the genes from the SFARI autism database and using the genes upon querying the DisGeNet portal. In addition to the genes reported by querying DisGeNet, we also used the genes reported in the literature for schizophrenia and depression, and for Parkinson’s disease. We found congruence in all cases.

To further test our hypothesis that these mental illnesses are *disorders of the nervous systems* and that removing the gene pool associated with them gives rise to overlapping tissues related to CNS functioning, we also queried DisGeNet about other non-neurological diseases. We found that in such cases, the predominance of maximally affected tissues was on tissues associated with peripheral vital bodily organs related to the disease, such as pancreas, kidney, liver, and colon transverse in colon cancer. Furthermore, several of these diseases had maximally affected heart-related tissues and whole blood. Other cases also revealed a predominance of peripheral organs. We lastly, interrogated the genome in relation to mitochondrial diseases and acquired PTSD. In these cases, we hypothesized and confirmed a mixture of tissues related to peripheral organs (for mitochondrial diseases) and the CNS (for PTSD).

In the case of mitochondrial diseases, the heart-related tissues were revealed as the most affected along with muscle-skeletal tissue. Furthermore CNS-related tissues were more affected by gene removal in mitochondrial myopathies (i.e., the amygdala and the anterior cingulate cortex), as compared to MELAS or mitochondrial disease. The common thread across all three types of mitochondrial-related disorders was the heart-related tissues. The case of acquired PTSD showed a mixture of CNS-related tissues, tissues related to bodily peripheral organs, and heart-related tissues. The kidney and pancreas were also affected in PTSD. When we examined the maximum Δλ for each disorder/disease under examination, we found that the basal ganglia was maximally affected in autism, unipolar depression, spinocerebellar ataxia, and PTSD), while the heart left ventricle tissue was maximally affected in depression, ataxia, cerebellar ataxia, gait ataxia and fragile X tremor ataxia syndrome (FXTAS). This result indicates overlap between the psychiatric mental illnesses and the neurological disorders. It also shows the importance of examining mental illnesses in a more systemic way that includes the autonomic nervous system of the PNS.

This test on non-neurological illnesses served as a control to show that removal of the genes associated with each disorder did have specificity with the disorder and yet a very different outcome when comparing the mental illnesses to the non-neurological disorders. Among the top tissues affected across non-neurological diseases, the pancreas was maximally affected by the removal of disease-associated genes in breast and colon cancer, while the liver was maximally affected in diabetes. The heart left ventricle was maximally affected across autoimmune disorders such as psoriasis, lupus systemic erythematosus, and irritable bowel disease (IBD). The heart was also maximally affected in hematologic neoplast, congenital heart disease, mitochondrial disease and MELAS, in contrast to the mitochondrial myopathies which showed the liver as the maximally affected tissue.

This exercise demonstrated that despite the stochastic nature of gene expression, upon removal and random recombination, there is convergence across psychiatric and neurological disorders, thus potentially rendering both as *disorders of the nervous systems*. In both cases, we found a strong prevalence of the CNS, but also found important differences in tissues from the PNS, including the heart and the muscle-skeletal tissues involved in both mental illnesses and neurological disorders. Because of these convergences, and the fact that there are treatments and accommodations to help persons with neurological disorders, it may be possible to leverage some of those types of bodily-based supports to help persons with mental illnesses. Behaviors that are described by observation to define mental illnesses can now be connected with underlying tissues involved in voluntary, involuntary, and autonomic function across the CNS and the PNS and mapped to the genome, thus closing the present gap between behaviors and genomics in the precision medicine knowledge network. In this sense, the present methods offer a new way to interrogate the genome and link tissues with behavioral phenotyping.

A surprising finding here is the potential contributions of peripheral structures and organs to mental illness. Tissues of the autonomic nervous systems were maximally impacted by the removal of the genes associated with these mental illnesses, as was the muscle-skeletal tissue among the top-ranked illnesses. Tissues associated with subcortical brain regions necessary for motor control, learning, adaptation, and coordination (basal ganglia and striatum) were highly impacted by the removal of the genes in both mental illnesses and neurological disorders, along with those tissues important for memory (hippocampus), emotion (amygdala) and regulation (hypothalamus). Surprisingly, we did not see cerebellum-related tissues among the most affected by the removal of the genes (even in ataxias) where we do know that the cerebellum plays a large role [20,21,22,23]. This was also the case in autism, where we know the cerebellum has been implicated [24,25].

Lastly, the autoimmune disorders that we examined had very different brain tissue patterns from the mental illnesses and neurological conditions but shared the heart-related tissues and the muscle-skeletal tissue. In this sense, the contributions from the peripheral systems to mental illnesses and to autoimmune disorders seem important. However, blood tissue marked a departure of neurological disorders from mental illnesses, as it was maximally affected in neurological disorders but not in the mental illnesses. Overall, these gene removals revealed surprising results that invite rethinking how we may want to describe, diagnose, and treat mental illnesses in general.

### Caveats and Future Directions

Although we found evidence that the mental illnesses and neurological disorders have remarkable overlap in the types of brain tissues that are maximally affected by the removal of their corresponding associated genes, we recognize that gene removal is a crude way to interrogate the human genome and its expression of the 54 tissues of the GTEx database. Future work will aim at developing more sophisticated methods to explore gene overexpression and to build simulations of the use of these methods in, e.g., combination with dynamic transcriptome evolution during neuronal differentiation in the development of cell lines from induced pluripotent stem cells. This will be important to move beyond a static approach and be able to assess asynchronous gene expression behaviors over time when cell lines differentiate into neuronal types. Full transcriptome dynamic interrogation longitudinally, over time, is now possible using these stochastic analyses in combination with the various data repositories featuring disease-associated genes.

The present work merely scratches the surface on possible new ways to interrogate the human genome in relation to diseases of all types (not just mental or neurological) in order to possibly build comparative models of outcomes *in tissues* that can be related to behavioral phenotypic manifestations of the clinical disorder. In this sense, the work presented here can help bridge the gap between behavioral description of a mental illness, or a neurological disorder, and its genetic underpinnings via the affected tissues. Combining this approach with the new wave of digital biomarkers that describe human behavior digitally at a microscopic level [9,26,27,28,29], using objective means and a finer level of granularity beyond naked eye detection, could help us redefine many psychiatric disorders and medical conditions under the precision medicine paradigm.

## 5. Conclusions

We here offer a new roadmap to reframe psychiatry using the precision medicine paradigm. The new stochastic approach can initiate the steps to connect behavioral phenotypic description from clinical observation and digital characterizations therein, with the underlying neurobiology of mental illnesses. Borrowing knowledge from neurology and brain science, it will be possible to shift psychiatry from an art to a quantitative objective science under the tenets of precision medicine by integrating all layers of the knowledge network. This would help design personalized targeted treatments utilizing the person’s genome, localizing the most-affected tissues defining central nervous system functions and distinguishing those from tissues related to vital organs for systemic functions. This new approach could potentially mark the beginning of a transformative era in mental health.

## 6. Patents

E.B.T. holds the US Patent “Methods and Systems for the Diagnoses and Treatments of Nervous Systems Disorders” combined in this paper as micro-movement spikes (MMS) data type and continuous gamma probability distribution family empirical estimation.

## Supplementary Materials

The following are available online at https://www.mdpi.com/2075-4426/10/4/144/s1, Figure S1: Outcome of genes’ expression on most affected tissues upon removing the Ataxia genes from the normative data., Figure S2: Outcome of genes’ expression on most affected tissues upon removing the FX and PD genes from the normative data., Figure S3: Outcome of genes’ expression on most affected tissues upon removing the Mitochondrial disease genes from the normative data, Figure S4: Outcome of genes’ expression on most affected tissues upon removing genes based on SFARI Autism scores., Figure S5: Information on gene CACNA1C common to the SFARI Autism set and the Ataxia Autosomal Dominant genes reported in the literature., Figure S6: Information on gene SCN2A common to the SFARI Autism set and the Ataxia Autosomal Dominant genes reported in the literature, Figure S7: Information on gene ATP1A3 common to the SFARI Autism set and the Ataxia Autosomal Dominant genes reported in the literature., Figure S8: Information on gene CCDC88C common to the SFARI Autism set and the Ataxia Autosomal Dominant genes reported in the literature., Figure S9: Information on gene ITPR1 common to the SFARI Autism set and the Ataxia Autosomal Dominant genes reported in the literature., Figure S10: Information on gene KCNJ10 common to the SFARI Autism set and the Ataxia Autosomal Recessive genes reported in the literature., Figure S11: Information on gene WWOX common to the SFARI Autism set and the Ataxia Autosomal Recessive genes reported in the literature., Figure S12: Information on gene GRID2 common to the SFARI Autism set and the Ataxia Autosomal Recessive genes reported in the literature., Figure S13: Information on gene LAMA1 common to the SFARI Autism set and the Ataxia Autosomal Recessive genes reported in the literature., Figure S14: Information on gene PEX7 common to the SFARI Autism set and the Ataxia Autosomal Recessive genes reported in the literature., Figure S15: Information on gene SYNE1 common to the SFARI Autism set and the Ataxia Autosomal Recessive genes reported in the literature., Figure S16: Information on gene CYP27A1 common to the SFARI Autism set and the Ataxia Autosomal Recessive genes reported in the literature., Figure S17: Information on gene SNX14 common to the SFARI Autism set and the Ataxia Autosomal Recessive genes reported in the literature., Figure S18: Information on gene RAB39B common to the SFARI Autism set and the Ataxia Autosomal Recessive genes reported in the literature., Figure S19: Information on gene CASK common to the SFARI Autism set and the X-Chromosome genes reported in the literature., Figure S20: Information on gene FMR1 common to the SFARI Autism set and the X-Chromosome genes reported in the literature., Figure S21: Information on gene SLC9A6 common to the SFARI Autism set and the X-Chromosome genes reported in the literature., Figure S22: Information on gene OPHN1 common to the SFARI Autism set and the X-Chromosome genes reported in the literature., Table S1: Genes common to the SFARI Autism set and the Ataxias and PD-early onset literature.

## Appendix A

TPM explanation from the site: “Transcripts Per Million (TPM) is a normalization method for RNA-seq, should be read as for every 1,000,000 RNA molecules in the RNA-seq sample, x came from this gene/transcript. For each transcript in the gene model, the number (raw count) of reads mapped is divided by the transcript’s length, giving a normalized transcript-level expression. The distribution of ambiguous reads (between transcripts of the same gene, or between different genes) is handled by OmicSoft’s RSEM implementation. The sum of ALL normalized transcript expression values is divided by 1,000,000, to create a scaling factor. Each transcript’s normalized expression is divided by the scaling factor, which results in the TPM value. Gene-level TPM’s are calculated by summing up the transcript-level TPM for each gene. In this scaling, the sum of all TPMs (transcript-level or gene-level) should always equal 1,000,000. For cells that have approximately the same number of transcripts-per-cell, the TPM expression values can be compared between these cells to estimate relative abundance. For a given sample, TPM values will linearly scale with FPKM values for genes or transcripts, but FPKM will not add up to 1,000,000, so TPM can also be thought as FPKM, scaled to sum to 1,000,000”.

Derivation of Maximum Likelihood Estimation of the rate parameter in the Exponential Distribution:

We estimate the likelihood $L\left(\lambda |x_{1},x_{2},\ldots ,x_{n}\right)$, where *x_i_* is the series of counts representing the gene expression on each given tissue and *i* ranges from 1 to *n*, the number of genes.

(A1) $$\begin{array}{cc} L\left(\lambda |x_{1},x_{2},\ldots ,x_{n}\right) & =\lambda e^{-\lambda x_{1}}\lambda e^{-\lambda x_{2}}\ldots \lambda e^{-\lambda x_{n}}\\ & =\lambda^{n}\left(e^{-\lambda x_{1}}e^{-\lambda x_{2}}\ldots e^{-\lambda x_{n}}\right)\\ & =\lambda^{n}\left(e^{-\lambda \left(x_{1}+x_{2}+\ldots +x_{n}\right)}\right) \end{array}$$

To obtain the maximum likelihood, we take the derivative of the likelihood in Equation (A1) and set it to 0 (since the derivative is 0 at the maximum likelihood value).

(A2) $$\frac{d}{d\lambda }L\left(\lambda |x_{1},x_{2},\ldots ,x_{n}\right)=\frac{d}{d\lambda }\lambda^{n}\left(e^{-\lambda \left(x_{1}+x_{2}+\ldots +x_{n}\right)}\right)$$

We take the log here because the derivative of the function and the derivative of the log of the function equals 0 at the same point. So, for the purposes of finding where the derivative is 0, the original function in Equation (A2) and the log of it are interchangeable.

(A3) $$\begin{array}{l} \frac{d}{d\lambda }\log\left(\lambda^{n}\left(e^{-\lambda \left(x_{1}+x_{2}+\ldots +x_{n}\right)}\right)\right)=\frac{d}{d\lambda }\log\lambda^{n}+\log\left(e^{-\lambda \left(x_{1}+x_{2}+\ldots +x_{n}\right)}\right)\\ \frac{d}{d\lambda }n\log\lambda -\lambda \left(x_{1}+x_{2}+\ldots +x_{n}\right)=0\\ n\frac{1}{\lambda }-\left(x_{1}+x_{2}+\ldots +x_{n}\right)=0\\ \lambda =\frac{n}{\left(x_{1}+x_{2}+\ldots +x_{n}\right)} \end{array}$$

Further, with this result in Equation (A3), we can obtain the maximum likelihood estimate of each *λ_j_*, given all the 56,146 genes expressed with some random value for each of the j=1:54 tissues.

## Appendix B

**Table A1:** Gene distributions used in the removal process and literature sources.

**Neurological**	**Number of Genes**	**Source**
Parkinson’s disease	17	Lit Review [14,16,17,18,19]
Ataxia Autosomal Recessive	70	Lit Review [11,12]
Ataxia Autosomal Dominant	46	Lit Review [11,12]
X-chromosome	6	Lit Review [11,12]
Fragile X (SFARI scores)	73 ^1^ (17,11,30,15)	SFARI Genes Module
Fragile X Syndrome	194	DisGeNet
FXTAS	62	DisGeNet
Ataxia	813	DisGeNet
cerebellar Ataxia	421	DisGeNet
gait Ataxia	159	DisGeNet
Progressive cerebellar Ataxia	134	DisGeNet
Spinocerebellar Ataxia	145	DisGeNet
**Neuropsychiatric (DSM)**	**Number of Genes**	**Source**
autism (SFARI scores)	906 ^1^ (144,216,468,78)	SFARI Genes Module
Autistic Disorder	1043	DisGeNet
schizophrenia	2697	Lit Review [30,31,32,33,34,35] and DisGeNet
Mental depression	1468	DisGeNet
depression Unipolar	641	DisGeNet
depression Bipolar	116	DisGeNet

^1^ SFARI scores for autism and FX genes were also used to assess each scored module separately. FXTAS stands for fragile X tremor ataxia syndrome.

**Table A2:** Genes associated with non-neurological diseases.

**Non-Neurological**	**Number of Genes**	**Source**
Colon Cancer	3669	DisGeNet
Diabetes Mellitus (non-insulin dependent)	3134	DisGeNet
Estrogen Receptor-Positive Breast Cancer	510	DisGeNet
Congenital Heart Disease	267	DisGeNet
Hematologic Neoplasm	827	DisGeNet
Systemic Lupus Erythematosus	1883	DisGeNet
Psoriasis	1308	DisGeNet
Irritable Bowel Syndrome	1483	DisGeNet
**Mixed**	**Number of Genes**	**Source**
Mitochondria	41	Lit Review [13]
Mitochondrial Myopathies	121	DisGeNet
Mitochondrial Diseases	284	DisGeNet
MELAS Syndrome	81	DisGeNet
PTSD	418	DisGeNet

MELAS (a form of dementia) stands for mitochondrial encephalopathy, lactic acidosis, and stroke-like episodes.

The data file name from the GTEx portal https://www.GTExportal.org/home/datasets used in this paper is GTEx_Analysis_2017-06-05_v8_RNASeQCv1.1.9_gene_median_tpm.gct.csv (accessed on 18 September 2020).

The data file name from the SFARI genes is located at https://gene.sfari.org/database/human-gene/ and named SFARI-Gene_genes_03-04-2020release_03-05-2020export.csv (accessed on 18 September 2020).

**Table A3:** The 13 top median-ranked tissues in descending order of Δλ value, the most-affected tissues upon removal of the SFARI genes (906) linked to autism from the human GTEx database in column 1. Ataxias, X-chromosome, fragile X, Parkinson’s disease and mitochondrial disease extracted from the literature and column 3 is the same as in column 1 while removing from the SFARI autism set 14 genes that overlap with ataxias and PD (see those genes listed in Supplementary Table S2). Tissues are grouped by CNS (brain and spinal cord in blue); muscle-skeletal (green), heart (pink) and peripheral organs (gray). SFARI autism (11/13 (84.6%) CNS, 1/13 (7.6%) heart and 1/13 (7.6%) peripheral organ); neurological disorders (10/13 (76.9%) CNS, 2/13 heart (15.3%) and 1/13 (7.6%) muscle-skeletal); SFARI autism without the overlapping genes from the neurological disorders (11/13 (76.9%) CNS, 1/13 (7.6%) heart and 1/13 (7.6%) peripheral organs).

SFARI Autism	Ataxias, X, FX, PD, Mitochondria	SFARI Autism without Overlapping Ataxia, PD Genes
Putamen Basal Ganglia	Hippocampus	Putamen Basal Ganglia
Substantia Nigra	Amygdala	Substantia Nigra
Amygdala	Substantia Nigra	Amygdala
Hippocampus	Heart Left Ventricle	Hippocampus
Caudate Basal Ganglia	Whole Blood	Caudate Basal Ganglia
Anterior Cingulate Cortex	Muscle Skeletal	Nucleus Accumbent Basal Ganglia
Nucleus Accumbent Basal Ganglia	Nucleus Accumbent Basal Ganglia	Anterior Cingulate Cortex
Hypothalamus	Putamen Basal Ganglia	Hypothalamus
Brain Cortex	Anterior Cingulate Cortex	Brain Cortex
Frontal Cortex	Brain Cortex	Frontal Cortex
Heart Left Ventricle	Hypothalamus	Spinal Cord
Spinal Cord	Heart Atrial Appendage	Heart Left Ventricle
Pancreas	Caudate Basal Ganglia	Kidney Cortex

**Table A4:** Most-affected tissues upon removal of the DisGeNet genes associated with autistic disorders (1043) from the human GTEx database (column 1); ataxia (813 genes) in DisGeNet (column 2) and FX (194 genes) in DisGeNet (column 3). Convergence between autism and neurological disorders is noted in the shaded tissues color coded as in Table A4, based on CNS, heart, and peripheral organs.

DisGeNet Autistic Disorders	DisGeNet Ataxia	FX DisGeNet
Substantia Nigra	Heart Left Ventricle	Hippocampus
Nucleus Accumbent Basal Ganglia	Amygdala	Whole Blood
Caudate Basal Ganglia	Hippocampus	Caudate Basal Ganglia
Putamen Basal Ganglia	Whole Blood	Substantia Nigra
Hippocampus	Putamen Basal Ganglia	Putamen Basal Ganglia
Amygdala	Substantia Nigra	Muscle Skeletal
Heart Left Ventricle	Anterior Cingulate Cortex	Nucleus Accumbent Basal Ganglia
Spinal Cord	Muscle Skeletal	Anterior Cingulate Cortex
Hypothalamus	Heart Atrial Appendage	Amygdala
Pancreas	Caudate Basal Ganglia	Brain Cortex
Heart Atrial Appendage	Nucleus Accumbent Basal Ganglia	Heart Left Ventricle
Anterior Cingulate Cortex	Brain Cortex	Kidney Medulla
Kidney Medulla	Hypothalamus	Frontal Cortex

**Table A5:** Overlapping genes between ataxias (dominant and recessive genes) and Parkinson’s disease with the genes from the SFARI portal. Scores in parenthesis refer to the scoring of the gene according to the SFARI site (see Methods for explanation on each category). Syndromic is (4). Supplementary Figures S5–S18 provide the GTEx violin plots of these gene expressions in the top-ranked tissues unveiled by our analyses. Supplementary Table S2 compiles additional information on the genes from various sources in the clinical literature.

SFARI Autism and Ataxia Dominant	SFARI Autism and Ataxia Recessive	SFARI Autism and (Early Onset) PD
CACNA1C (1)	KCNJ10 (2)	RAB39B (3)
SCN2A (1)	WWOX (2)	
ATP1A3 (3S)	GRID2 (3)	
CCDC88C (3)	LAMA1 (3)	
ITPR1 (3)	PEX7 (3)	
	SYNE1 (3S)	
	CYP27A1 (4)	
	SNX14 (4)	

**Table A6:** Most-affected tissues upon removal of the DisGeNet genes associated with different types of ataxias, color coded by CNS (brain and spinal cord), heart-related, muscle-skeletal, and peripheral vital organ for systemic functioning. Predominance of CNS is evident, followed by heart-related and muscle-skeletal and peripheral organs.

Gait Ataxia	Spinocerebellar Ataxia	Cerebellar Ataxia	Progressive-C Ataxia
Heart Left Ventricle	Caudate Basal Ganglia	Heart Left Ventricle	Liver
Substantia Nigra	Brain Amygdala	Substantia Nigra	Heart Left Ventricle
Hippocampus	Substantia Nigra	Hippocampus	Whole Blood
Muscle Skeletal	Putamen Basal Ganglia	Amygdala	Heart Atrial Appendage
Caudate Basal Ganglia	N Accumbens BG	Whole Blood	Putamen Basal Ganglia
N Accumbens BG	Ant Cingulate Cortex	Putamen Basal Ganglia	Substantia Nigra
Whole Blood	Hippocampus	Hypothalamus	Hippocampus
Brain Amygdala	Heart Atrial Appendage	Ant Cingulate Cortex	Brain Amygdala
Kidney Cortex	Hypothalamus	Caudate Basal Ganglia	Muscle Skeletal
Putamen Basal Ganglia	Brain Cortex	N Accumbens BG	Kidney Cortex
Ant Cingulate Cortex	Frontal Cortex	Brain Cortex	Caudate Basal Ganglia
Hypothalamus	Heart Left Ventricle	Kidney Cortex	N Accumbens BG
Heart Atrial Appendage	Kidney Cortex	Muscle Skeletal	Ant Cingulate Cortex

**Table A7:** Most-affected tissues upon removal of the DisGeNet genes associated with schizophrenia (2697) from the human GTEx database; depression genes (1468); unipolar depression genes (641); and bipolar depression genes (116). Convergence between schizophrenia and depression is high, with maximally affected CNS tissues (10/13), followed by heart-related and muscle-skeletal tissues. Unipolar and bipolar depression also show systemic effect of vital peripheral organs (N stands for Nucleus, Ant for Anterior, and BG for Basal Ganglia).

Schizophrenia	Depression	Unipolar Depression	Bipolar Depression
Amygdala	Heart Left Ventricle	Putamen Basal Ganglia	Hippocampus
Putamen Basal Ganglia	Heart Atrial Appendage	Caudate Basal Ganglia	Putamen Basal Ganglia
Heart Left Ventricle	Hippocampus	Hippocampus	Amygdala
Hippocampus	Muscle Skeletal	Substantia Nigra	Caudate Basal Ganglia
Substantia Nigra	Putamen Basal Ganglia	Kidney Cortex	Muscle Skeletal
Caudate Basal Ganglia	Caudate Basal Ganglia	Amygdala	Substantia Nigra
Ant Cingulate Cortex	Amygdala	Heart Left Ventricle	Spinal Cord
N Accumbens BG	Pancreas	Kidney Medulla	Esophagus Muscularis
Hypothalamus	Kidney Medulla	Brain Cortex	Adrenal Gland
Muscle Skeletal	Substantia Nigra	N Accumbens BG	Frontal Cortex
Pancreas	Kidney Cortex	Colon Transverse	Minor Salivary Gland
Brain Cortex	Ant Cingulate Cortex	Ant Cingulate Cortex	Colon Sigmoid
Frontal Cortex	N Accumbens BG	Heart Atrial Appendage	Ant Cingulate Cortex

**Table A8:** Most-affected tissues upon removal of the DisGeNet genes associated with colon cancer (3669) from the human GTEx database; irritable bowel syndrome genes (1483); congenital heart disease genes (267); and hematologic neoplast genes (827). Color code as in previous tables.

Colon Cancer	Irritable Bowel Syndrome	Congenital Heart Disease	Hematologic Neoplast
Pancreas	Heart Left Ventricle	Heart Left Ventricle	Heart Left Ventricle
Whole Blood	Pancreas	Heart Atrial Appendage	Whole Blood
Stomach	Kidney Cortex	Kidney Cortex	Liver
Adrenal Gland	Liver	Whole Blood	Heart Atrial Appendage
Colon Transverse	Muscle Skeletal	Kidney Medulla	Pancreas
Prostate	Heart Atrial Appendage	Substantia Nigra	Muscle Skeletal
Small Intestine T	Kidney Medulla	Hypothalamus	Kidney Cortex
Spleen	Whole Blood	Prostate	Hippocampus
Minor Salivary Gland	Adrenal Gland	Hippocampus	Kidney Medulla
Fallopian Tube	Adipose Viseral Oment	Muscle Skeletal	Esophagus Mucosa
EBT Lymphocytes	Esophagus Mucosa	Adipose Visceral O	EBT Lymphocytes
SkinNoSunExposed S	Colon Transverse	Minor Salivary Gland	Substantia Nigra
Liver	Stomach	Pancreas	Putamen Basal Ganglia

**Table A9:** Most-affected tissues upon removal of the DisGeNet genes associated with lupus systemic erythematosus (1883) from the human GTEx database; psoriasis genes (1308); breast cancer (510); and diabetes genes (3134). The top half of the highest-ranked tissues show no convergence with CNS-related tissues found in the mental illnesses and neurological disorders interrogated in this work. Instead, heart-related tissue, muscle-skeletal tissue and tissues related to peripheral vital organs for systemic functioning are found. The bottom half of the top Δλ-ranked tissues are a mixture of tissues in peripheral bodily organs and brain-related tissues. The latter are from motor control, coordination, and adaptation subcortical areas and from emotion, memory, and regulatory areas. Color code as in previous tables.

Systemic Lupus Erythematosus	Psoriasis	Breast Cancer	Diabetes
Heart Left Ventricle	Heart Left Ventricle	Pancreas	Liver
Whole Blood	Liver	Heart Left Ventricle	Pancreas
Liver	Whole Blood	Heart Atrial Appendage	Heart Atrial Appendage
Heart Atrial Appendage	Muscle Skeletal	Kidney Medulla	Heart Left Ventricle
Pancreas	Heart Atrial Appendage	Muscle Skeletal	Kidney Medulla
Muscle Skeletal	Pancreas	Whole Blood	Kidney Cortex
Kidney Cortex	Kidney Cortex	Ant Cingulate Cortex	Putamen Basal Ganglia
Hippocampus	Putamen Basal Ganglia	Esophagus Mucosa	Substantia Nigra
Kidney Medulla	Caudate Basal Ganglia	Kidney Cortex	Hippocampus
Esophagus Mucosa	Adipose Visceral O	Adrenal Gland	Caudate Basal Ganglia
EBT Lymphocytes	Substantia Nigra	Esophagus Muscularis	Amygdala
Substantia Nigra	Esophagus Mucosa	Hypothalamus	N Accumbens BG
Putamen Basal Ganglia	Hypothalamus	Putamen Basal Ganglia	Hypothalamus

**Table A10:** Most-affected tissues upon removal of the DisGeNet genes associated with mitochondrial disease (284) from the human GTEx database; mitochondrial myopathies genes (121); mitochondrial encephalopathy, lactic acidosis, and stroke-like episodes (MELAS) (81); Post-Traumatic Stress Disorder genes (418).

Mitochondrial Disease	Mitochondrial Myopathies	MELAS	PTSD
Heart Left Ventricle	Liver	Heart Left Ventricle	Caudate Basal Ganglia
Muscle Skeletal	Heart Left Ventricle	Liver	Kidney Medulla
Pancreas.	Muscle Skeletal	Kidney Cortex	Kidney Cortex
Heart Atrial Appendage	Hippocampus	Pancreas	Pancreas
Kidney Cortex	Whole Blood	Heart Atrial Appendage	Putamen Basal Ganglia
Whole Blood	Ant Cingulate Cortex	Kidney Medulla	Heart Left Ventricle
Kidney Medulla	Brain Amygdala	Putamen Basal Ganglia	Hypothalamus
Ant Cingulate Cortex	Substantia Nigra	Esophagus Mucosa	Substantia Nigra
Putamen Basal Ganglia	Heart Atrial Appendage	Brain Amygdala	Heart Atrial Appendage
N Accumbens BG	Putamen Basal Ganglia	Adipose Visceral Omen	N Accumbens BG
Adrenal Gland	Spinal Cord	Artery Coronary	Hippocampus
Hippocampus	Pancreas	Stomach	Colon Sigmoid
Caudate Basal Ganglia	Hypothalamus	Colon Traverse	Amygdala

**Table A11:** Most-affected tissue in each disease/disorder according to the maximum Δλ value (See Figure 13 in the main text).

Disease/Disorder	Maximally Affected Tissue
Autism DisGeNet	BrainSubstantiaNigra
ASD NoAtaxias	BrainPutamenBasalGanglia
Autism SFARI	BrainPutamenBasalGanglia
Schizophrenia	BrainAmygdala
Depression DisGeNet	HeartLeftVentricle
Bipolar Depression	BrainHippocampus
Unipolar Depression	BrainPutamenBasalGanglia
ATAXIA DisGeNet	HeartLeftVentricle
SpinoCerebellar ATAXIA	BrainCaudateBasalGanglia
Cerebellar ATAXIA	HeartLeftVentricle
ProgressiveCerebellar ATAXIA	Liver
Gait ATAXIA	HeartLeftVentricle
FAXTAS	HeartLeftVentricle
FX	BrainHippocampus
PTSD	BrainCaudateBasalGanglia
Diabetes	Liver
BreastCancer	Pancreas
ColonCancer	Pancreas
HematologicNeoplast	HeartLeftVentricle
CongenitalHeartDisease	HeartLeftVentricle
IBD	HeartLeftVentricle
LupusSystemicErythomatosus	HeartLeftVentricle
Psoriasis	HeartLeftVentricle
MItochondriaDisease	HeartLeftVentricle
MELAS	HeartLeftVentricle
MitochondriaMyopathies	Liver
